# Feasibility and acceptability of a universal screening and referral protocol for gender-based violence with women seeking care in health clinics in Dadaab refugee camps in Kenya

**DOI:** 10.1017/gmh.2017.18

**Published:** 2017-10-30

**Authors:** A. Vu, A. L. Wirtz, S. Bundgaard, A. Nair, G. Luttah, S. Ngugi, N. Glass

**Affiliations:** 1 Department of Emergency Medicine, Johns Hopkins Medicine, Baltimore, Maryland, USA; 2 International Health, Johns Hopkins University Bloomberg School of Public Health, Baltimore, Maryland, USA; 3 Department of Epidemiology, Johns Hopkins University Bloomberg School of Public Health, Baltimore, Maryland, USA; 4 Health and Women's Empowerment and Protection, International Rescue Committee, New York, New York, USA; 5 Johns Hopkins University School of Nursing, School of Nursing, Baltimore, Maryland, USA

**Keywords:** Gender-based violence, interventions, refugee, screening

## Abstract

**Background.:**

Gender-based violence (GBV) is both a global public health problem and violation of human rights. Refugees and internally displaced persons experience an increased risk of GBV and health outcomes associated with GBV are often exacerbated in conflict settings.

**Methods.:**

A mixed methods study to examine the feasibility and acceptability of universal screening for GBV in a refugee population in the Dadaab refugee camp of Kenya, using the ASIST-GBV from January to July 2015.

**Results.:**

Of 9366 women offered screening at International Rescue Committee health clinics, about 89% (*n* = 8369) female refugees consented to participate. Only 15% of the potentially eligible population could participate in GBV screening because of the ongoing struggle to identify private space in the clinics. Over 85% of women reported being ‘willing’ or ‘very willing’ to participate in GBV screening; 96% felt they had a good or very good experience with the screening protocol. Qualitative findings stressed the importance of securing a room/space in the busy clinic is critical to universal screening with referral to safe and confidential services for survivors.

**Conclusions.:**

The findings suggest that the evidence-based ASIST-GBV is both feasible to implement and acceptable to both providers and women seeking care. Universal GBV screening and referral is an effective way for health care and service providers in humanitarian settings to assist survivors of GBV.

## Background

Gender-based violence (GBV) is both a global public health problem and a violation of human rights. The Interagency Standing Committee defines GBV as, ‘…harmful act that is perpetrated against a person's will and that is based on socially ascribed (i.e. gender) differences between males and females’ (IASC, 2015). Globally, an estimated 35% of women have experienced physical, sexual intimate partner violence (IPV) or non-partner sexual violence in their lifetimes (WHO, 2013). Among conflict-affected populations, at least one in five (21.4%) women report sexual violence during their lifetime (Vu *et al.* 2014). This figure is an underestimation of GBV, given that it does not capture the full range of GBV and that GBV remains largely under-reported and unrecognized among refugee and internally displaced person (IDP) populations. Multiple factors that contribute to vulnerability to GBV among refugee and IDP populations during and post-displacement include but are not limited to: build up of military and armed groups; collapse of social and family support structures; breakdown of security and police protection; insecure physical infrastructure; financial instability; and strained relationships with host community. Serious and potentially life-threatening health outcomes that are associated with GBV are exacerbated by the lack of infrastructure, support services, and reduced access to health care in conflict settings, and limited funding allocation (Tanabe *et al.* 2015).

Global advocates and organizations have called upon humanitarian practitioners to (1) establish clear reporting, monitoring, referral, and evaluation mechanisms to expand access to assistance and (2) to provide comprehensive health care that is easily accessible to refugee and displaced populations. Despite recent efforts to increase disclosure and strengthen care, under-reporting of GBV persists as survivors remain unaware of rights and available services for GBV or are reluctant to self-report and seek services. Barriers of disclosure to service providers and local authorities include shame and fear of discrimination, lack of knowledge about the availability of services, and lack of confidence in GBV services (Wirtz *et al.* 2013; Wirtz *et al.* 2014).

To collaboratively strengthen efforts by trained health care providers to support disclosure and refer survivors to appropriate services, the Johns Hopkins ‘Assessment Screen to Identify Survivors Toolkit for Gender Based Violence’ (ASIST-GBV) was developed and validated with female and male refugees and IDPs in Ethiopia, Colombia, and Uganda (Wirtz *et al.* 2013; Wirtz *et al.* 2014; Vu *et al.* 2016; Wirtz *et al.* 2016). The findings from initial evaluations of the ASIST-GBV in these diverse settings have demonstrated the potential to allow skilled providers in humanitarian settings to confidentially and efficiently screen survivors for GBV, improve rates of confidential disclosure, increase referrals to available services such as health, psychosocial, and protection services. Moreover, use of GBV screening may support surveillance efforts by humanitarian and host government institutions by identifying the types (i.e. physical violence, sexual violence, threats and coercive control) of GBV experienced and the related health, psychosocial and protections needs of survivors in camps/settlements and may ultimately inform programming and policies. This paper advances our previous work by presenting the findings of the feasibility and acceptability of GBV screening using the ASIST-GBV tool offered to women seen at the International Rescue Committee's (IRC) primary health facilities in the Dadaab refugee camps in Kenya.

## Methods

This study was conducted over a 6-month period from January to July 2015 and utilized a mixed-methods design to assess the feasibility and acceptability of implementation of GBV screening in refugee health clinics in Dadaab, Kenya. Quantitative assessments included: (1) use of the ASIST-GBV by trained providers with all consenting adult female refugees living in the Dadaab refugee camps and who sought health services at the participating IRC clinics, and (2) exit interviews among female refugees who completed GBV screening. Qualitative research included: (1) in-depth interviews among a subset of refugee women who had been identified through screening as a survivor of GBV who had been referred to and accessed services, and (2) focus group discussions among health care and other service providers using ASIST-GBV, providing referral services, or otherwise involved in the implementation of GBV screening. To inform the community about the screening program, community outreach workers also informed members of the refugee population as part of their work with IRC. IRC has a very active program in the Dadaab refugee camps that utilize community outreach programming to address issues related to maternal and child health, community health, mental health programming and follow up care.

### Setting and participants

GBV screening was implemented by trained IRC health and service staff in the IRC-managed health clinics in the Hagadera and Kambios refugee camps of Dadaab, Kenya, from January to July 2015. The Dadaab refugee camps, the largest refugee settlement globally, are comprised of 5 camps and host 276 945 refugees (UNHCR, 2016). As of 2016, the refugee population was primarily composed of Somali refugees (94.9%), though a minority were refugees Ethiopia (4.4%) and <1% from the Democratic Republic of Congo, South Sudan, and Burundi (UNHCR, 2016). All female refugees aged 15 years and older presenting alone to the health clinics were eligible for GBV screening. Women were excluded from GBV screening if they were accompanied, unable to provide informed consent, or if screening could not be conducted in a private location.

### ASIST-GBV adaptation

The ASIST-GBV is a brief 7-item screening tool designed to confidentially identify for referral a range of GBV, including threats of violence, physical violence, sexual violence and exploitation, forced pregnancy, forced abortion, and forced marriage in the last 12 months (Vu *et al.* 2016). ASIST-GBV was adapted to local contexts through discussions with local IRC staff and subsequently translated into Somali language as Somalia refugees are the vast majority of camp population (UNHCR, 2016). Cognitive testing methods and back-translation were then employed to minimize response errors and errors in translation. Pilot testing of the ASIST-GBV was then conducted on this final translated version of the tool. For eligible women that did not speak Somali or English, camp interpreters were trained to confidentially support the health care providers.

### Recruitment and data collection procedures

Trained health providers privately offered screening to female refugees who attended the IRC health clinics as part of routine health services. Support of translators was provided, as appropriate. Prior to screening, eligible participants underwent an informed consent process that provided information about the project, including the purpose, risks, benefits, and safety procedures to ensure protection of research participants. No personal identifiers were collected. Because written consents could potentially enable linkage of a participant's name to the study in an otherwise anonymous study, verbal consent scripts were used to ensure participant confidentiality.

### Exit interviews

After completion of screening and clinical care and prior to leaving the clinic, Somali-speaking research assistants who had not been involved in screening invited a subsample of women at each clinic to participate in brief exit interviews. Exit interviews (*N* = 101) were conducted in private. These 15-item surveys intended to measure various aspects of acceptability, including: understanding of the purpose of screening; feelings/nervousness prior to, during, and after screening; opinions about confidentiality, privacy, and safety of the screening process; how staff managed discomfort when screening; acceptability of having a health professional ask screening questions; and overall acceptability of the screening process.

### Qualitative research

In July 2015, in-depth interviews were conducted among the pool of refugee women who completed screening, screened positive for GBV, and had been referred for additional services (*N* = 19). Women attending the IRC referrals were invited to meet with qualitative interviewers at a later time and date to provide feedback on the screening and referral process. Participants were purposively sampled based on age, other demographics, and cultural ethnic representation. Purposive sampling was also used to enroll health care and service providers who had implemented screening or had received referrals from screening within the Dadaab camps to participate in focus group discussions (*N* = 24) to understand their experience with the screening and referral program. Both in-depth interviews and focus group discussions followed a semi-structured interview/discussion guide.

### Qualitative analysis

The audio recordings of the qualitative interviews were translated and transcribed by bilingual (Somali-English speaking) research assistants in Dadaab, Kenya. The Johns Hopkins (JHU) team member coded each transcript for data analysis using pre-specified codes and following a thematic approach. Topical codes were applied to allow quotations to be sorted according to interview guide domains and open interpretive coding was utilized to identify and analyze any emerging themes observed within and between topical areas. This allowed for in-depth exploration of additional emerging themes in the participants’ narrative responses. Quotations were selected to highlight themes that emerged from the analysis. Additional supporting quotes are provided in the appendix and referenced in the following text as: Additional-file.

### Quantitative analysis

Quantitative assessments (ASIST-GBV screening questionnaires and exit interviews) were completed on paper questionnaires. IRC staff entered responses from paper questionnaires into a database that was created in EpiInfo (version 3.5.4, Centers for Disease Control and Prevention, Atlanta, GA). The data were imported into Stata/SE (version 11, StataCorp, College Station, TX) for analysis by JHU. Evaluation of the feasibility of screening was analyzed through process indicators, including the number of women screened, number screened positive for past-year GBV, and referred for care. Descriptive analysis was also conducted for the analysis of acceptability measured by exit interviews. Data from GBV Information Management System (GBVIMS) was used to visual the trend in referrals before and after GBV screening was implemented (GBVIMS Steering Committee, 2010).

### Human subjects protection

Participant confidentiality was strictly enforced per IRB JHU protocols for all phases of data collection. Participants aged 15 years or older were included in the study as an ethical mandate given their particular vulnerability to GBV. The consent forms were developed and tested in consultation with IRC's Health and Women's Empowerment and Protection (WPE) service providers. All participants completed informed consent prior to screening and qualitative participants, as well as health and WPE service providers, completed informed consent prior to participating in qualitative research. Comprehensive clinical and psychosocial services are available to survivors of all age ranges in Dadaab. Training for providers who implemented the screening and referral protocol included confidentiality, safety planning, and discussion of health needs of participants in addition to any training previously received as part of employment with IRC. The training and implementation process included the referral pathways established with local GBV and child protection programs and services.

## Results

### Feasibility

Though it was intended that all women who attended the selected health clinics would be routinely offered screening, about 15% of potential participants were offered screening (9366/64 212) due to providers’ preexisting work load and limited private space in which screening could be conducted. This was reflected in the focus group discussions with providers, in which the feasibility of finding a space to privately conduct the screening was a common concern (Additional-file, quote 02):

*“There is no special room for this. We are just using rooms that were meant for other purposes. If possible special room for the screening is good.”* – Service provider manager

Table 1 shows the basic demographic information of female refugees who were offered GBV screening. Of the 9366 female refugees who were offered screening, 8369 (89%) consented to participate in screening.

**Table 1.** Demographics of refugee females who were offered GBV screening, January–July 2015

Age	*N* = 9266	%
15–19	1080	11.5
20–29	4116	43.9
30–39	2447	26.1
40–49	428	4.6
50–59	61	0.7
60 and older	1134	12.1
Years displaced	*N* = 9266	
1–2	399	4.3
3–5	2216	23.7
6–10	1890	20.2
11–15	1527	16.3
16–20	972	10.4
>20	2362	25.2
Consented	*n* = 9266	
Yes	8369	89.4
Refused	997	10.6

Table 2 displays the types of past-year GBV reported among women. The most common form of recent (past year) GBV was physical violence (1.4%), followed by threat of GBV (1.0%) and sexual exploitation (0.7%). Out of a total 8369 screened participants, 213 (2.5%) were identified as a survivor of at least one type of GBV in the past 12 months.

**Table 2.** Types of violence reported from GBV screening

Types of violence reported	*n*/*N*	%
Threat of violence	81/8356	1.0
Physical	113/8353	1.4
Forced sex	41/8355	0.5
Sexual exploitation	54/8357	0.7
Forced Pregnancy	28/8354	0.3
Forced abortion	25/8339	0.3
Forced Marriage	27/8343	0.3
Any past year experience of GBV	213/8369	2.5

**Fig. 1.**
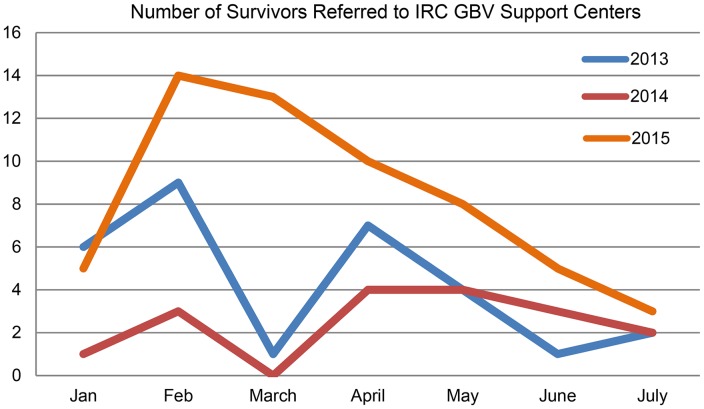
Survivors referred from health clinics to IRC support center during ASIST-GBV screening period compared with previous year

According to the data captured in the GBVIMS, of the 234 GBV survivors that attended referral services at an IRC support center available for all five camps in Dadaab, 58 (24.9%) had been referred from IRC clinics where GBV screening had been implemented and 33 (57%) of these women were direct referrals from the GBV screening clinic protocol. Overall, the GBVIMS observed an increase in the number of health center referrals during screening, from January to July 2015, relative to the same period in 2014, suggesting overall increases in community awareness of GBV and uptake of services associated with the implementation of GBV screening. The IRC confirmed this constituted a significant increase (241%) in referrals compared with the previous year (2014). Figure 1 displays trends over time.

Overall, the health and service providers who participated in focus group discussions noted that GBV screening could be successfully implemented in health care centers with appropriate training, flexibility and resources. Providers implementing screening found that explaining the screening and responding to individual questions, which were often similar across participants, during the consent, process was adding substantial time to the screening process and overall health visit. As a result, providers modified the process by providing a group information session on GBV screening and consent in the waiting area during which people could ask questions prior to moving into a private room for their clinical visit and screening. Once the participant was in the private room where screening would take place, they completed the remainder of consent process and participated in screening. This process of providing a group education session increased general awareness about GBV and substantially reduced time needed to answer frequently asked questions for each participant (Additional-file, quote 01). Providers indicated that no negative effects were associated with this process and also reported that routine and universal screening demonstrated to the community that they were not targeting any specific individuals, thereby reducing risk of stigma related to disclosing GBV and accessing services. As noted, the ASIST-GBV protocol (informed consent, GBV screening and referral) was implemented during the confidential clinical consultation:

*“Basically what happens in the antenatal clinic is one of the key entry points where we do the GBV screening. You find every morning we have health education whereby we talk to the mothers regarding the screening process, tell them what is entitled in the screening process in terms of what GBV is. Then from there we have a one on one session with the mothers…That means that a patient enters into the room, one, we do all the ANC profiles, in case she has any other problem we normally treat, then we also introduce the aspect of the screening whereby we get the consent, tell them what it is entailed. There on if she accepts we do the screening, if she doesn't accept we thank her.”* – Service provider

### Acceptability

Quantitative measures of acceptability were derived from the exit interviews conducted among 101 female refugees who had undergone GBV screening. The age distribution of those participating in the exit interviews was approximately similar to the age distribution of the total sample that completed the GBV screening. Table 3 displays responses to the exit interview surveys. Over 85% reported being willing or very willing to participate in GBV screening, with similar percentage reporting being not really or not at all nervous about participating in GBV screening. Over 75% reported that it was acceptable for health providers to ask about experiences of GBV. About 94% felt safe responding to GBV questions and over 89% thought that GBV screening would help women get access to needed services.

**Table 3.** Exit interviews responses

Item	*N*	%
What was reason for GBV screening in health center	*n* = 100	
1. The staff likes to ask questions	3	3.0
2. The staff wants to know if violence is happening in the camp	18	17.8
3. The staff needs to report this to their supervisors	21	20.8
4. The staff wants to know if someone experienced violence and offer them options for care.	58	57.4
Willingness to participate in GBV screening	*n* = 101	
1. Very willing	53	52.5
2. Willing	35	34.7
3. Not very willing	8	7.9
4. Not at all willing to participate	5	5.0
Nervous about being asked about GBV?	*n* = 101	
1. Very nervous	11	10.9
2. A little nervous	6	5.9
3. Not really nervous	17	16.8
4. Not at all nervous	67	66.3
Why nervous	*n* = 28	
1. Worried that others may overhear	1	3.6
2. Worried about what the staff would think	1	3.6
3. Was not sure what would happen	16	57.1
4. Did not really understand the purpose of being asked these questions	10	35.7
Did staff do or say anything to make you feel less nervous?	*n* = 27	
1. No	1	3.6
2. Yes, they explained the purpose of the screening/questions	12	42.9
3. Yes, they told me all of my information would be kept private	3	10.7
4. Yes, they told me that they could offer me additional services if I have experienced GBV	3	10.7
5. Yes, we spoke privately so no one overheard	8	28.6
Are questions too sensitive?	*n* = 101	
No	77	76.2
Yes	24	23.8
Is it acceptable for providers to ask about recent GBV?	*n* = 98	
No	21	20.8
Yes	77	76.2
Why not acceptable	*n* = 21	
1. There is nothing they can do to care for gender-based violence	6	28.6
2. People who experience violence have no rights	1	4.8
3. The violence will continue anyway	4	19.0
4. Don't know	4	19.0
5. No response	6	28.6
Why is it acceptable for provider to ask about GBV?	*n* = 80	
1. They can provide health care for violence	22	27.5
2. They can help to prevent violence	27	33.8
3. It is important to know that the staff are concerned	3	3.8
4. They can provide psychosocial/ psychological care for violence	28	35.0
Feel your responses are overheard?	*n* = 101	
No	97	96.0
Yes	4	4.0
Feel your responses are kept private	*n* = 101	
No	19	18.8
Yes	78	77.2
Don't know	4	4.0
Feel safe responding to GBV question	*n* = 101	
No	6	5.9
Yes	95	94.1
Think GBV screening will help women to access services	*n* = 100	
1. It is very helpful to them	62	61.4
2. Somewhat helpful	28	27.7
3. Not very helpful	7	6.9
4. Not at all helpful	3	3.0
Rate your experience being screened for GBV	*n* = 100	
1. Very good	49	48.5
2. Pretty good	48	47.5
3. Pretty bad	1	1.0
4. Terrible	2	2.0

Qualitative interviews and focus groups among referred GBV survivors and service providers complement these quantitative findings and provide in-depth understanding of acceptability of GBV screening implementation as it relates to three key components of GBV screening and referral: consent to GBV screening, implementation of GBV screening, referral process to services, and uptake of referral services.

#### Consent to GBV screening

Participants reported mixed feelings when they were first offered the opportunity to participate in GBV screening, with some indicating that they were initially nervous and others reporting they were not worried at all:

*“I was nervous because I thought they will expose my secret about the rape…. I feared that they would tell other especially my family. I did not want them to know I was raped… I was happy when they hide my secret.”* – Survivor, 28 years

The implementation of GBV screening and referral protocol within the clinics was spread by word-of-mouth among female refugees. In this process, participants also described the importance of confidentiality and privacy during the process, which appeared to encourage others to participate (Additional-file, quote 05):

*“Everyone comes here but their information is kept private, so I thought they will keep also my secret. I asked my friend and she told me they don't share your information with others…that is why I come to hospital she told me not fear.”* – Survivor, 28 years

Health and service providers described some initial resistance to GBV screening during the health care visits when the GBV screening implementation began. However, this concern was reduced over time as communities became more aware that GBV screening was a routine part of health care services at the clinics. Outreach workers noted that women were more willing to disclose GBV because they were now aware of GBV services, referral options and quality of care available in the camp.

*“My friend says ‘you have initiated a nice program because initially we did not have somewhere to expose our feelings.’ The reason is people feel that they have now understood the program.”* – Outreach worker

#### Implementation of GBV screening

Initial concerns among some participants were related to perceptions about the professionalism of health providers, especially confidentiality, as well as the availability to secure private space for implementation of GBV screening. These concerns dissipated over time as reported by women seeking care (Additional-file, quote 06-07):

*“[What did the staff do to try to make you feel comfortable during screening?] They advised and told them how safety, peace is important and [they] support the people who have experienced GBV and if the person is depressed to counsel the person so that she feels okay and their morale come up.”* – Survivor, 30 yrs.

The content and length of ASIST-GBV screening tool were reportedly acceptable to participants. Participants also noted that GBV screening within the health clinic demonstrated that the staff cared about them and their experiences. Participants highlighted the importance of compassion of providers in the implementation of GBV screening.

*“We can answer the [screening] questions. When someone share their problems they feel better. It shows that someone cares about you and they talk with you about the problem.”* Survivor, 26 years

Implementing GBV screening with young women who are often accompanied by their mothers or other female family members to the clinic are served as an initial challenge to providers. Mothers often preferred to participate in the clinical consultation with their daughter; however, this is likely a barrier for the daughter to safely report GBV, as it is possible that the mother is involved or knows about GBV (Additional-file, quote 08). Providers and young women used strategies to ensure confidentiality and privacy, including allowing only patient-provider clinical consultations and explaining to the mother or other family member the importance of confidentiality and privacy. Providers were able to offer young women referrals for GBV services without the mother's knowledge through the private clinical consultation.

Service providers also noted the challenge of high turnover of health and other social service staff and the need for ongoing GBV trainings to reach new staff and refresher trainings for remaining staff. Health and service providers also highlighted the importance of having enough staff and ensuring trained, dedicated staff members were available to maintain quality of implementing the ASIST-GBV protocol.

*“Maybe in the future time if you plan, if we have a more modernized place for receiving women, or recruiting more staff in the health post so that the one's giving out the information and then one's also attending the client, I think that one may improve the [screening].”* – Service provider

#### Uptake of referral services

Referrals to the IRC-managed GBV support center, where clinical care and counseling were provided, was offered to all participants who screened positive for GBV. Additional services were also available at this center, including educational and economic empowerment programs. At the minimum, those who went to the support center received counseling services. Information on available GBV resources was also given to women who screened negative.

*“[As far you can remember, what kind of options did the staff provide to you in terms of getting treatment or care for your experience of gender- based violence?] I came here several times while I was very angry with my husband because of his other wife then they referred me to counseling session, they counseled me and I left here while I was relieved and laughing.”* – Survivor, 35 years

Participants generally reported feelings of comfort and safety upon accessing the services at the support center (Additional-file, quote 09):

*“When I was here for the first I was crying and badly injured. I was very depressed but when I was sent to training I felt comfortable and I left the support centre when I was happy because I felt safe and secure and there are people who are there to support us.”* – Survivor, 30 yrs.

Women participants also described challenges in accepting referrals and services. These were primarily related to distance to reach the support center and the need for childcare when leaving home to access services (Additional-file, quote 10–11).

Overall, service providers felt that despite slower initial uptake of screening by refugees attending the clinic, initial apprehensions subsided and screening allowed women to safely disclose GBV and providers to support women who disclosed and refer them to services. Providers reported that clinic-based group education on GBV, the universal screening, and the referral program delivered by trained and skilled providers had begun to make positive changes in social norms so that women were able to safely disclose GBV and accessing needed services (Additional-file, quote 12–13):

*“This screening activity has improved our referral way of the clients. Initially we were depending on our outreach staff to go to the blocks and then take cases. They are the ones who used to bring these clients to the support center. This time we are trying… We are getting more survivors, more clients, simply because of the screening activity at the health post.”* – Outreach worker

## Discussion

Refugee populations are at increased risk for GBV in the settings of armed conflict, as well as during transit/displacement and in the camp/settlement setting. Loss of secure housing, limited economic opportunities, lack of security, and family disruption may increase vulnerability to opportunistic violence as well as IPV (Christian *et al.* 2011; Wirtz *et al.* 2013; Hossain *et al.* 2014; Wirtz *et al.* 2014). Guidelines have been established to support the development of a minimum package of services to prevent and respond to GBV in humanitarian settings (United nations High Commissioner for Refugees, 2003; United Nations Population Fund, 2012; IASC, 2015). Screening for GBV is an evidence-based strategy that can support disclosure of GBV by survivors and allow for medical, psychosocial support, and referrals to appropriate GBV and other support services. ASIST-GBV provides a brief screening tool that has been validated in multiple countries for use among refugee and displaced populations (Wirtz *et al.* 2013; Vu *et al.* 2014; Wirtz *et al.* 2014; Vu *et al.* 2016; Wirtz *et al.* 2016). This study furthers the previous work to evaluate GBV screening and referral protocol as an acceptable and feasible strategy to ensure that survivors of past-year GBV receive necessary care. The implementation GBV screening in clinical care also has the potential to raise community awareness about available GBV services. Over a 6-month period, more than 9000 female refugees (aged 15 years and older) were offered screening and referrals using the ASIST-GBV protocol with nearly 90% acceptance. Health care and service providers as well as women who disclosed GBV expressed an overall positive experience with the implementation of GBV screening.

Provider acceptance of routine, universal screening improved the implementation period as they became more comfortable with the ASIST-GBV protocol. The time required to deliver the 7-item questionnaire was only 3–4 min when providers become proficient. Acceptance by providers improved as they gained confidence after explaining the relationship between GBV and health and the importance of accessing confidential services to improve health and safety. As implementation continued in the clinics, women began to communicate and endorse the confidentiality of the GBV screening intervention to other women in the camps. There was initial concern of conducting the screening and referrals with Somali women seeking care because of confidentiality issues within the refugee community. However, as Somali providers conducted the clinic-based group education on GBV while patients were waiting to be seen, the process was better understood, acceptance rates increased, and the screening and referral protocol was more efficiently integrated into the clinical consultation. Both providers and participants reported that universal screening may assist in changing social norms for disclosure and accessing GBV service in the traditionally conservative Somali population. Participants reported that they had informed neighbors or family members about GBV services available through the health clinics. More women seeking care were noted to request GBV screening as part of their clinical consultation because of the increased awareness of services available for GBV.

Most of the challenges in feasibility of GBV screening occurred early in implementation. The largest obstacle to implementation of universal screening was the lack of private spaces to maintain privacy within busy clinics. As a private space is mandatory for screening, this resulted in the inability to offer screening to all women during situations when space was limited in the camp clinics. Thus, while the majority of those offered GBV screening were willing to participate, only 15% of the potentially eligible population could participate in GBV screening because of the ongoing struggle to identify private space in the clinics. Identifying and dedicating space for providers to complete clinical consultations including GBV screening is critical to future universal screening with referral to safe and confidential services for women.

Although the providers were generally positive about the GBV screening intervention, the addition to their already heavy clinical and administrative workload proved to be a challenge. On an average day, each participating health clinic receives about 90 patients within a 7-h period. Security threats during the GBV screening implementation in Dadaab refugee camps, which became more common during the final 3 months of the program, also reduced clinic hours of operation, further restricting the availability of screening and services. Consequentially, a smaller number of women were offered GBV screening than anticipated. Given the workload challenge, the implementation of GBV screening focused on the last 12 months (as opposed to lifetime experience of GBV) in order to concentrate limited resources and services with GBV survivors who were more likely to need immediate care.

The workload challenge highlights the importance of having a women-centered approach to prevent and respond to GBV. A women-centered approach focuses on non-judgmental compassion care, confidentiality, and a team-based approach where all staff is trained on GBV and health, confidentiality and privacy, universal screening and referrals to available services. Training and mentorship for the multidisciplinary team approach (e.g. physicians, nurses, and community health workers) to implement the GBV screening intervention may reduce the stress and burden of having only a few staff trained to implement the protocol. Further, having the entire team trained may reduce the impact of provider turnover and the need for additional training sessions for clinic staff. New staff could be trained and mentored by previously trained staff.

Strategic modifications in the implementation process, such as conducting clinic-based GBV awareness through group education for women in waiting areas served to inform the target population of the ASIST-GBV protocol, was perceived by clinic staff to reduce the time needed by providers to respond to questions posed by women on the reasons for GBV screening. Clinic-based group education and universal screening and referrals also have the potential to reduce women's concern of being stigmatized when disclosing GBV and accessing referrals.

Additional implementation challenges were associated with uptake of referrals, which was reflected by the decrease in attended referrals reported by the GBVIMS in later months of screening. In particular, the main challenge noted was the distance women needed to travel to access the IRC managed support center, relative to the health clinics where the referrals were made. In response, service providers recommended that, where possible, future GBV screening implementation includes some basic and initial psychosocial support at the clinics by trained providers so that survivors have immediate access to needed services while they obtain access to the IRC support center. Further, some women who received referrals may not utilize the services immediately, and given the relatively short implementation (6 months) of the GBV screening intervention, we may not have captured service access related to all referrals provided.

Finally, the proportion of female refugees who disclosed any type of GBV in the past year was approximately 2.5%, which is well below the estimated 20% past-year prevalence of any type of GBV reported in other studies in similar settings (Vu *et al.* 2014). There are several factors contributing to this low proportion. Dadaab refugee camps are predominantly composed of refugees from Somalia. The conservative Somali cultural background may prevent female refugees from coming forward to disclose their GBV experiences as noted in our qualitative studies among Somali refugees living in Ethiopia (Wirtz *et al.* 2013). Additionally, it is important to note that this was a feasibility and acceptability study that was implemented in a relatively short period of time (6 months) with only 15% of women seeking care in health clinics completing the ASIST-GBV protocol. If more time was afforded to implementation and evaluation, providers will likely continue to gain confidence and skills in the universal implementation of GBV screening and women seeking care may feel confident in disclosing GBV and access needed services.

## Limitations

The study has limitations. The main limitation was our inability to conduct universal screening and referral among the entire population of female refugees, as discussed above. However, the ethical responsibility of health care and service providers to confidentially and safely implement screening and referral protocols was the priority. Further, the busy clinics and heavy workload for providers, as well as multiple languages requiring skilled translation, made it challenging for providers to prioritize implementing the GBV screening intervention with every eligible female patient. Another limitation was that we were not able to determine the proportion of survivors who accessed referral services that were offered to them. We did attempt to triangulate uptake of referral through data from GBVIMS as a proxy, though this does not fully determine effectiveness screening and referrals. Finally, it is important to also note that implementation research was not a population-based sampling of female refugees within the camp. Therefore, no estimations of prevalence in the camp population should be drawn from these data.

## Conclusion

The findings suggest that the evidence-based ASIST-GBV is both feasible to implement and acceptable to both providers and women seeking care. Universal GBV screening and referral is an effective way for health care and service providers in humanitarian settings to assist survivors of GBV. The universal implementation of GBV screening has the potential to create a confidential environment where survivors can disclose GBV; ensure women-centered and competent care and offer referrals based on individual needs and wishes of survivors; and increase awareness of GBV and available services among women and the community, thereby potentially changing norms of stigma and discrimination against the survivor. Future research of GBV screening in humanitarian settings should further examine the impact of screening on disclosure, utilization of GBV services, safety, health and repeat violence.

## Supplementary material

For supplementary material accompanying this paper visit http://dx.doi.org/10.1017/gmh.2017.18. click here to view supplementary material
