# Supplementary material for: Feasibility and acceptability of a universal screening and referral protocol for gender-based violence with women seeking care in health clinics in Dadaab refugee camps in Kenya
Source: Glob Ment Health (Camb). 2017 Oct 30;4:e21. doi: 10.1017/gmh.2017.18 (PMC5719473; doi:10.1017/gmh.2017.18)
Supplement: Supplementary file 1 [file S2054425117000188sup001.docx]

***Feasibility and Acceptability of a Universal Screening and Referral Protocol for Gender-Based Violence with Women Seeking Care in Refugee Health Clinics in Dadaab Camp, Kenya***

***Additional file***

**Table 1** Additional relevant quotations from refugee participants in in Dadaab Camp, Kenya, stratified by topic discussed in the results of manuscript. All quotations are marked with a sequential number (left column) to locate the associated text in the manuscript.

| **Feasibility:** | |
| --- | --- |
| No. | Quotation |
| 01 | *Initially [prior to group education in waiting area] it was very difficult for them to accept actually. It was time wasting. They were not understanding what exactly what is this being introduced and why they are being asked about their personal life or what is happening in their family and what benefit would they get from that screening...[after group education implementation] They’ve been accepting and right now as we speak it’s like we talk about it like any other topic, they’re not scared.* – Service provider |
| 02 | *The biggest problem with us has been confidentiality. We have very little space to do everything and the culture here is in such a way that people move freely. If there’s one patient in the room the others will not fear to come in. You might be in the middle of a discussion and six people just walk in and they have to stop, you talk to them again, convince them to sit.” –* Service provider |
| 03 | *I find women relate more to women than women to men. They’ll open up to a fellow woman than to a fellow man”* – Service provider |
| 04 | *“The screeners should all be female, because what we are screening are all female…Previously we mixed, but later we have realized that men are not suitable. We have changed. They are all women.”* – Service provider manager |

| **Acceptability** | |
| --- | --- |
| No. | Quotation |
| 05 | *I was nervous because maybe somebody would have negative attitude towards me. I just fear people will hear I had such experience… I think after talking to them and I was counseled I felt okay. I said to myself that it’s better to talk than to be silent.” -* Survivor, 26 years |
| 06 | *No, I did not worry at all because I was questioned by girls. I say what I wanted to say. I was happy to say my problems since I had hope that I will be helped.”* – Survivor, 28 years |
| 07 | *That was in a private place no one was near us. It was me and the interviewer.”* - Survivor, 28 years |
| 08 | *“She [the young patient] will tell you ‘my mother is the one who met with the man who married me, I don’t know him, he’s an old man’ or ‘it’s somebody who raped me last month’. You’ll find there’s a story behind that young girl who is being escorted… They [young participant] will always sneak in on their own and they will come and tell you I don’t want to be seen here. Talk to me fast and I go… You give them the [referral] card and then they will arrange. Still they will tell you I’ll come and go with that because when my mother sees me with her now the incentive staff she won’t have any problem. Or I’ll go myself. The moment you give them that card she will hide it or she will leave it there then she’ll tell you tomorrow morning ‘I’ll pass here, if you give me my card then go to the hospital’.” –* Service provider |
| 09 | *They [IRC staff] helped me get medicine after I was referred although the side I was hit still hurt. I got treatment I am better now.” -* Survivor, 30 yrs. |
| 10 | *I did not participate in any because I was pregnant walking from here to home is a problem.” –* Survivor, 26 years |
| 11 | *I have small children at home to take care of every morning I wake up early in the morning to go to market to look money to buy food. I come back in the evening so I was very busy I did not get chance to back. –* Survivor, 28 years |
| 12 | *It may also happen that some people have experienced GBV long time ago but they never had the opportunity to come and report their case to the GBV. Of course they were not even aware or they never had that information. This time, when a person is screened, she is given a lot of information about the support center GBV program and what they do. The person sometimes will feel happy and maybe try to share her experience even if it's like sometime back. The person will still have the pleasure to tell you about her experience. In that way, now I can say screening also has given women an opportunity to also sensitize the community and give them information about the GBV office and services that are provided from the GBV office.” –*Service provider |
| 13 | *Also that glass of conservativeness has been broken … At least there’s some cracks… at least people now can open up a little bit. Also the level of awareness has gone up because the more people talk about it, the more it becomes normal for people to speak out when they’re hurt. I think the screening has done some good things.” –*Service provider |
